# WHIM Syndrome: First Reported Case in a Patient of African Ancestry

**DOI:** 10.1155/2023/3888680

**Published:** 2023-02-06

**Authors:** Jinal Gandhi, Michelle H. Lee, Lynsie Adams, Tara Shrout Allen, Julie Li, Camille Vanessa Edwards

**Affiliations:** ^1^Section of Hematology and Medical Oncology, Department of Medicine, Boston University, Boston Medical Center, Boston, Massachusetts, USA; ^2^Department of Pathology, Moffitt Cancer Center, Tampa, FL, USA

## Abstract

**Background:**

Warts, hypogammaglobulinemia, infections, and myelokathexis (WHIM) syndrome is a rare, primary immunodeficiency syndrome characterized by warts, hypogammaglobulinemia, immunodeficiency, and characteristic bone marrow features of myelokathexis. The pathophysiology of WHIM syndrome is due to an autosomal dominant gain of function mutation in the CXCR4 chemokine receptor resulting in increased activity that impairs neutrophil migration from the bone marrow into the peripheral blood. This results in bone marrow distinctively crowded with mature neutrophils whose balance is shifted towards cellular senescence developing these characteristic, apoptotic nuclei termed myelokathexis. Despite the resultant severe neutropenia, the clinical syndrome is often mild and accompanied by a variety of associated abnormalities that we are just beginning to understand. *Case Report*. Diagnosis of WHIM syndrome is incredibly difficult due to phenotypic heterogeneity. To date, there are only about 105 documented cases in the scientific literature. Here, we describe the first case of WHIM syndrome documented in a patient of African ancestry. The patient in question was diagnosed at the age of 29 after a comprehensive work-up for incidental neutropenia discovered at a primary care appointment at our center in the United States. In hindsight, the patient had a history of recurrent infections, bronchiectasis, hearing loss, and VSD repair that could not be previously explained.

**Conclusions:**

Despite the challenge of timely diagnosis and the wide spectrum of clinical features that we are still discovering, WHIM syndrome tends to be a milder immunodeficiency that is highly manageable. As presented in this case, most patients respond well to G-CSF injections and newer treatments such as small-molecule CXCR4 antagonists.

## 1. Introduction

Warts, hypogammaglobulinemia, infections, and myelokathexis (WHIM) syndrome is a rare, primary immunodeficiency, first described in 1964 and later recognized in 2003 as an autosomal dominant disorder caused by a gain of function mutation in the chemokine receptor CXCR4 [1, 2]. CXCR4 and its ligand CXCL12 regulate the release of white blood cells (WBCs) from the bone marrow into the blood. In WHIM syndrome, increased CXCR4/CXCL12 signaling leads to the retention of fully mature, apoptotic neutrophils within the bone marrow (myelokathexis) which clinically manifests as severe neutropenia with or without lymphopenia and monocytopenia. Panleukopenia may result in low serum immunoglobulins and increased susceptibility to bacterial oto-sinopulmonary, skin and soft tissue infections, and treatment-refractory human papillomavirus (HPV)-induced warts [2]. Recurrent infections can have irreversible sequelae, such as bronchiectasis and hearing loss [3]. The mechanism of increased HPV infection severity is not well understood, but there is a known higher risk of malignant transformation. In addition, there is a similar risk of uncontrolled EBV and EBV-related lymphoproliferative diseases [4]. Despite severe neutropenia, infections in WHIM syndrome tend to be milder than those observed in patients with other primary immunodeficiencies as the neutropenia may be rapidly overcome by G-CSF, acute stress, or severe infection. Other clinical features of WHIM syndrome include cerebellar malformations and cardiac malformations such as ventricular septal defects (VSDs) and tetralogy of Fallot, which occur in approximately 7% of patients [3].

Due to considerable genotypic and phenotypic heterogeneity, WHIM syndrome remains a difficult diagnosis to make. Less than 40% of patients will have all four classic features and a corresponding CXCR4 mutation on presentation [5]. About half of the reported cases are children and roughly 60% are female. Race is reported in 42% of the known WHIM syndrome cases, none of which included patients of African ancestry. There have been reports of suspected WHIM syndrome in a cohort of Nigerian women; however, diagnostic evidence was never collected. Likewise, there is one reported case from South Africa but no race information was included [5]. Here, we describe a case of WHIM syndrome in a patient of African ancestry who presented with both classic and novel disease features.

## 2. Case Presentation

The case is of a 29-year-old female of African descent from Haiti who was referred to hematology for severe neutropenia. Notably, she had a history of cardiovascular malformation (ventricular septal defect) surgically repaired at the age of 5 and recurrent skin and oto-sinopulmonary infections (sinusitis, otitis media, and pneumonia) since childhood resulting in progressive hearing loss and chronic bronchiectasis. She also reported anosmia and recurrent blisters of the philtrum, likely herpetic lesions, although no such lesions were present at the time of our examination. Of note, her HSV-1 antibodies were positive on serology. The patient reported no history of tobacco smoking, alcohol use, or substance abuse. Her family history was notable for a brother with recurrent infections but no formal diagnosis of primary immunodeficiency. There was no other known family history of hematologic disorders, immunodeficiency, or malignancy. Family members had been offered genetic testing but to date, no specimens have been sent in for evaluation. Six years prior to her current presentation, she underwent a bone marrow biopsy to evaluate incidentally-discovered neutropenia. The biopsy showed a largely normal marrow with trilineage hematopoiesis, erythroid hypoplasia, and plasmacytosis without light chain restriction. An aspirate could not be performed, and no cytogenetic or molecular testing was available.

**Figure 1:**
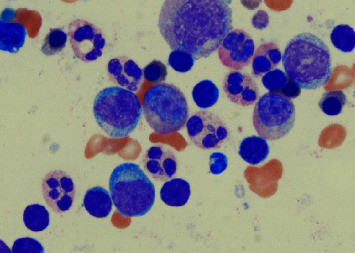
Bone marrow biopsy.

On the day of presentation to the hematology clinic, she appeared well. She had no abnormal facies, skin lesions, adenopathy, or organomegaly. Laboratory examination was notable for bicytopenia with a total white blood cell count of 2.1 (reference range: 4.0–11.0 × 10^3^/*μ*L), absolute neutrophil count (ANC) of 1.1 (reference range: 1.8–7.0 × 10^3^/*μ*L), absolute lymphocyte count 0.7 (reference range: 1.1–3.5 × 10^3^/*μ*L), absolute monocyte count 0.3 (reference range: 0.2–0.9 × 10^3^/*μ*L), and hemoglobin of 7.9 (reference range: 11.8–16 g/dL) (Table 1). Their platelet count was within normal limits and her renal and liver functions were normal. Immunoglobulin G, A, and E levels were high (Table 1). Vaccination responses were appropriate for diphtheria, tetanus, mumps, and rubeola while response to Hemophilus influenza vaccine was suboptimal. Additional work-up including HIV, EBV, CMV, HSV, varicella, and hepatitis B and C serologies were all negative. A routine work-up for anemia revealed no abnormalities. Vitamin B12, folate, and copper levels were within normal limits. Serum protein electrophoresis, serum immunofixation, and serum free light chain assay did not reveal monoclonal proteins [6] (Table 1). Iron studies revealed ferritin of 8 ng/mL and transferrin saturation of 5.6%, consistent with iron deficiency anemia. Peripheral blood smear showed dysmorphic neutrophils containing thin cytoplasmic connections between nuclear lobes (Figure 1). No immature WBCs or blasts were seen. Red cell and platelet morphology were normal. Given these findings on peripheral smear and flow cytometry, a bone marrow aspirate and biopsy was performed to rule out a primary hematologic disorder. The biopsy was notable for myelokathexis classically described as myeloid hyperplasia with right shift and nuclear vacuolization of some neutrophils along with altered lobation of nuclei connected by long, thin chromatin fragments (Figure 1). There were no dysplastic features. Next-generation sequencing was notable for a frameshift mutation of the CXCR4 gene located on chromosome 2q22 with the following mutation: S342^*∗*^ (c. 1025 C > G). This is a novel pathogenic variant that has not yet been described. A diagnosis of WHIM syndrome was made given the classic pathologic feature of myelokathexis, CXCR4 gain of function gene mutation, and clinical history of cardiovascular malformation, recurrent infections, and neutropenia.

The patient started long-term daily G-CSF injections at our pharmacy's maximum safe dose of 300 mcg. G-CSF dose continues to be titrated to obtain a neutrophil count of 500–1500 cells/microliter. To complete disease-specific screening, she was referred to obstetrics and gynecology for vaginocervical HPV screening and to dermatology for cutaneous HPV screening with monitoring. Pap smear was normal, and a plan for routine surveillance every three years was made. HPV vaccines were also administered. The patient was also offered genetic counseling. Additionally, she was seen by otolaryngology for anosmia. Work-up was unremarkable, including a normal brain MRI and no evidence of Kallmann syndrome. Finally, the patient started intravenous iron dextran for iron deficiency anemia given prior reported gastrointestinal side effects to oral iron. Since starting G-CSF therapy, she has had monthly visits with hematology for clinical evaluation and complete blood counts. To date, the patient remains well without additional skin or oto-sinopulmonary infections. She had minor upper respiratory tract symptoms due to COVID-19 infection. The patient recovered as an outpatient without sequelae, despite being unvaccinated or receiving any targeted treatment.

## 3. Discussion

Given the small cohort of reported WHIM cases to date, several unknowns remain. As such, this case highlights several important features. First, WHIM syndrome may arise de-novo without any prior family history (in around 33% of cases) and about 60% of patients present without warts or hypogammaglobulinemia [2]. In addition, there may be other associated clinical characteristics that we have yet to identify, such as the unexplained anosmia seen in our patient.

The mainstay of treatment for WHIM syndrome includes WBC mobilizing agents such as G-CSF with an ANC goal >500. G-CSF both stimulates bone marrow production of myeloid cells and promotes neutrophil release by enhancing neutrophil elastase, which interrupts CXCR4-CXCL12 signaling that is known to trap neutrophils within the marrow [7]. Notably, G-CSF only increased ANC but not absolute lymphocyte or monocyte counts. Monthly intravenous immunoglobulin is considered for patients with hypogammaglobulinemia, and prophylactic antibiotics are generally only recommended in the face of recurrent infections [4]. Our patient did not require the latter. As in our case, several WHIM syndrome patients do not have hypogammaglobulinemia despite having severe B cell lymphopenia. The cause remains unknown at this time [5]. Observational studies suggest that many patients with WHIM syndrome have a particularly severe reduction in CD27+ memory B cells, so although they have a normal capacity to produce antibodies against antigens, including from vaccines, they fail to maintain antibody production [8].

There is no cure for WHIM syndrome. Enrollment in clinical trials is recommended if available. There are currently two small-molecule CXCR4 antagonists being studied, plerixafor and mavorixafor. Plerixafor was studied in two phase 1 trials which showed that absolute neutrophil, lymphocyte, and monocyte counts were elevated in the peripheral blood. Open-label studies of longer-term use (6 months to 1 year) showed a reduction in the frequency of infections, reduced frequency of warts, clearance of HPV, and improved quality of life. No serious adverse effects were reported. However, the use of this drug is severely limited given its short half-life (about 5 hours) and cost [9]. Mavorixafor is a similar agent with a longer half-life (23 hours). Phase 2 clinical trial outcomes are promising, showing durable increases in WBC subtypes, decreased annual infection rates, and reduction in HPV-associated cutaneous warts [6]. Other CXCR4 antagonists are currently under investigation but have not yet been studied in WHIM patients [10–12].

Moreover, curative strategies with allogeneic stem cell transplantation and gene therapy have been used in selected patients. The largest pooled cohort of patients with WHIM syndrome treated with hematopoietic stem cell transplantation (HSCT) includes seven pediatric patients from around the world [13]. Six of the patients survived; one patient died from graft rejection. Five patients achieved complete resolution of all hematologic and immunologic manifestations of WHIM syndrome. Most notably, one patient had delayed lymphocyte recovery more than 1.4 years after HSCT and another developed EBV-associated post-transplant lymphoproliferative disease requiring chemotherapy and ultimately donor anti-EBV specific cytotoxic T-lymphocyte infusion. The oldest patient to have received HSCT was of age 20; this patient's disease was complicated by myelofibrosis which was subsequently cured with HSCT [14]. Although this could be an option for our patient, the risks associated with HSCT should be weighed against a manageable, nondisabling burden of disease. Finally, the CRISPR/Cas9 gene-editing system is currently being deployed for investigation in this setting. While no tenably curative treatment exists yet, WHIM syndrome can be manageable with safe treatment options, and new pathways for discovery offer greater hope moving forward.

## 4. Conclusion

WHIM syndrome is a rare disease where the pathologic finding of myelokathexis is invariably seen but the clinical phenotype is markedly heterogeneous. Therefore, a high index of suspicion is needed, and each individual case contributes additional knowledge regarding clinical presentation and associated features. To our knowledge, our case is the first reported in a patient of African descent and the finding of otherwise unexplained anosmia is also unique. In keeping with the pathophysiology of the disease involving a gain of function mutation in CXCR4, our patient has responded well to G-CSF. Novel agents targeting the CXCR4 pathway, such as plerixafor and mavorixafor, are currently under investigation. Given the long-term sequelae and increased risk of malignancy in patients with WHIM syndrome, additional clinical research is warranted to develop a pathobiology-inspired approach to eventually cure this disease.

This slide is presented at 1000x magnification with Wright Giemsa stain. This is the patient's bone marrow sample which revealed classic features associated with WHIM syndrome. There are normal trilineage hematopoiesis, myeloid hyperplasia with right shift, and the hallmark features of myelokathexis: nuclear vacuolization of some neutrophils along with altered lobation of nuclei connected by long, thin chromatin fragments. The features are suggestive of apoptotic nuclei due to aberrant bone marrow retention.

## Figures and Tables

**Table 1:** Pertinent lab values at initial presentation.

Pertinent lab data	Values	Reference range
WBC	**1.3 K/*μ*L (L)**	4.0–11.0 × 10^3^/*μ*L
Absolute neutrophil count	**0.2 K/*μ*L (L)**	1.8–7.0 × 10^3^/*μ*L
Absolute lymphocyte count	**1.1 K/*μ*L (L)**	1.1–3.5 × 10^3^/*μ*L
Absolute monocyte count	0.3 K/*μ*L	0.2–0.9 × 10^3^/*μ*L
Absolute eosinophil count	0	0.0–0.6 × 10^3^/*μ*L
Absolute basophil count	0	0.0–0.1 × 10^3^/*μ*L
Hemoglobin	**7.9 g/dL (L)**	11.8–16.0 g/dL
Hematocrit	**27.5% (L)**	36.0%–47.0%
MCV	**79 (L)**	80–97 FL
RDW	**19% (H)**	32.0–36.0 g/dL
Platelet count	179 K/*μ*L	150–400 g/dL
Iron	**20** **mcg/dL (L)**	51–146 mcg/dL
TIBC	357 mcg/dL	240–450 mcg/dL
Ferritin	**8** **ng/mL (L)**	10–109 ng/mL
IgA	**523 mg/dL (H)**	70–400 mg/dL
IgG	**1804 mg/dL (H)**	700–1600 mg/dL
IgM	185 mg/dL	46–304 mg/dL
IgE	**260 mg/dL (H)**	0–200 IU/mL

^*∗*^Abnormal values appear in bold where H = high and L = low.

## Data Availability

The citations used to support the findings of this study are included within the article in the “References” section.
